# Tuberculosis presenting as Anterior Mediastinal Mass: A Case Report

**DOI:** 10.31729/jnma.8508

**Published:** 2024-03-31

**Authors:** Simran Pradhan, Image Khadka

**Affiliations:** 1 Department of Research and Development, National Open College, Lalitpur, Nepal; 2 Department of Anaesthesiology, Patan Academy of Health Sciences, Lagankhel, Lalitpur, Nepal

**Keywords:** *case reports*, *mediastinal mass*, *tubercular meningoencephalitis*, *tuberculosis*

## Abstract

The mediastinum, located between the pleural sacs, has three compartments. The anterior mediastinum spans anteriorly from the sternum to the pericardium and brachiocephalic vessels posteriorly.Common lesions in this area include thymomas, lymphomas, teratomatous neoplasms, and thyroid masses. A mediastinal mass in the setting of tuberculous meningoencephalitis is an uncommon presentation of tuberculosis. We present a case of a 20-year-old girl with fever and headache diagnosed with tuberculous meningoencephalitis. A thorough workup revealed an anterior mediastinal mass, histopathologically diagnosed as tubercular in origin. Treatment involved surgery and antituberculosis therapy. Tuberculosis can manifest uniquely, and an isolated mediastinal mass, especially in an immunocompetent individual, is unusual. Treatment typically involves a combination of antimicrobial medications, and in some cases, surgical intervention may be necessary to address complications or persistent masses. This case emphasizes the importance of considering tuberculosis as a diagnosis when a patient presents with a mass in the anterior mediastinum.

## INTRODUCTION

The anterior mediastinum accounts for 50% of mediastinal masses.^1^ While two-thirds are benign, about 59% in the anterior compartment are malignant.^2^ Tuberculosis can manifest in various ways and affect any part of the body; however, presenting solely as an isolated mediastinal mass, as observed in our patient without parenchymal lesions, is rare.^3^ Despite closely resembling malignant lesions on radiology, tuberculosis can be effectively treated with better outcomes compared to other common mediastinal masses.^4^ This case emphasizes the importance of considering tuberculosis in such presentations. Treatment combining medical and surgical approaches can yield a favorable prognosis, as evidenced in our case.

## CASE REPORT

A 20-year-old girl presented to Dhulikhel hospital, Nepal in February 2021 with complaints of fever, headache and vomiting over the past 2 months. The fever was intermittent, more pronounced in the evening, with no associated cough, chest pain, shortness of breath, hemoptysis, or weight loss. On examination, she was afebrile and both vital signs and systemic examinations were unremarkable. However, neurological examination revealed neck stiffness, but Kernig and Brudzinski signs were negative. There was no peripheral lymph node enlargement, facial puffiness, or neck vein prominence.

A complete blood count showed normal values, including hemoglobin (12.6 gm/dL), total count of 7300 cells/dL with neutrophil 81%; lymphocytes 15%; monocytes 1%; and eosinophils: 0%), and platelets count of 450×10^3^/dL). Clinical biochemistry, including renal and hepatic functions, yielded normal results. HIV ELISA was negative. Chest radiography illustrated soft tissue enlargement within the paratracheal stripe. In light of meningoencephalitis suspicion, a lumbar puncture was performed, and cerebrospinal fluid (CSF) analysis indicated tuberculous meningoencephalitis. Gram stain showed no organisms, and CSF adenosine deaminase was 8. Magnetic Resonance Imaging (MRI) head was done which revealed ring-enhancing lesions and edema suggesting tuberculoma. She was diagnosed with a tuberculoma and initiated on antitubercular therapy. A craniotomy was initially planned, but due to the presence of a mass in the anterior mediastinum and the potential need for cardiopulmonary bypass, the operation was postponed.

**Figure 1:**
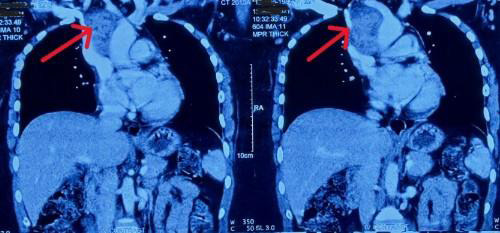
Preoperative CECT of chest showing mass in the anterior mediastinum (red arrow).

Further evaluation of the anterior mediastinal mass was carried out, and a contrast-enhanced computed tomography (CECT) scan of chest was done for further characterization of the mass which revealed an ill-defined, heterogeneously enhancing lesion in the anterior mediastinum, suggestive of a thymic lesion or confluent, necrotic lymph nodes (possibly tubercular or metastatic) (Figure 1).

**Figure 2:**
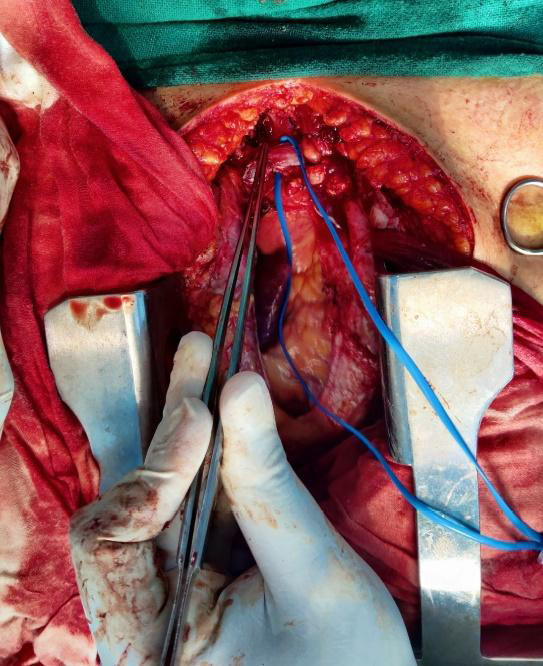
Per-operative finding showing anterior mediastinal mass.

Consequently, she underwent a sternotomy with debulking of the anterior mediastinal mass. Per operative findings include a mass of 4x4 cm in the anterior mediastinum, with adhesions extending into innominate vein, trachea, aorta and superior vena cava. There was an enlarged thymus gland involving both lobes, while the heart, pericardium, and lungs appeared normal (Figure 2).

The excised mass was sent for histopathological examination and Gene Xpert. The biopsy revealed necrotizing granulomatous inflammation of tubercular origin. Gene Xpert indicated very low *Mycobacterium tuberculosis* detection without rifampicin resistance. A four-drug combination therapy with anti-tuberculosis drugs was continued for 9 months, leading to the patient's clinical improvement without any recurrence of the mediastinal mass or symptoms. On follow-up for 9 months, she did not have any significant issues.

## DISCUSSION

Tuberculosis is a globally prevalent disease that inflicts suffering and death upon millions of people. It constitutes a major public health challenge in Nepal. In 2018/19, the estimated tuberculosis prevalence in Nepal was 416/100,000, with approximately 117,000 people living with tuberculosis in the country.^5^ The mortality was also determined to be 3.1 times higher than the previous estimates.^5^ Despite this substantial impact, the diagnosis and treatment of tuberculosis continue to pose significant challenges in Nepal.

Tuberculosis can manifest in diverse ways, but presenting as an isolated mediastinal mass, as observed in our patient without parenchymal lesions, is unusual.^3^ However, such presentations are not uncommon in immune-compromised patients.^4^ Conditions such as HIV infection are associated with an increased frequency of mycobacterial infections, particularly in lymph nodes.^6^ Although the lungs are the most common site for tubercular infection, it can affect virtually any part of the body. Mediastinal lymph nodes are a common site of primary infection in children, and mediastinal lymphadenopathy on chest radiographs is frequently observed. There have been very few case reports of adult mediastinal tuberculosis in the literature.^7,8^ However, a study done in India revealed a strikingly elevated number (14.28%) of mediastinal tuberculosis cases, possibly attributed to the prevalence of the disease in underdeveloped and developing countries like ours.^9^

Tuberculosis in different anatomical locations presents with distinct signs, symptoms, and investigative findings. Patient with mediastinal tuberculosis usually present with constitutional features such as fever, weight loss, lack of appetite, and night sweats.^10^ Our patient exhibited some constitutional symptoms, including fever and headache; however, there was an absence of any chest symptoms.Common differential diagnoses for an anterior mediastinal mass include thymoma, lymphoma, teratomatous neoplasms, thyroid masses, vascular masses, lymph node enlargement due to metastases or granulomatous disease, and pleuropericardial and bronchogenic cysts.

These diagnoses help guide appropriate additional imaging or other diagnostic procedures and therapeutic approaches. However, a definitive diagnosis requires histopathological examination of tissue from the mass. In our patient, histopathology revealed epithelioid cell granulomas with necrosis. The granulomatous lesion may be attributed to sarcoidosis, tuberculosis, or fungal infections.^10^ Sarcoidosis is characterized by non-necrotizing granulomatous inflammation, while both tubercular and fungal granulomas may exhibit necrosis.

Surgical resection might be needed for the majority of anterior mediastinal masses, as was performed in our patient. The patient underwent a sternotomy with mass debulking. The excised mass was sent for histopathological examination which revealed necrotizing granulomatous inflammation, likely of tubercular origin. With the prompt commencement of Anti-Tubercular Treatment (ATT), successful management of these patients is attainable. Our patient was started on four-drug combination therapy with anti-tuberculosis drugs which was continued for 9 months, leading to the patient's clinical improvement without any recurrence of the mediastinal mass or symptoms.

The complex presentation of tuberculosis, in which the patient presented with features of tuberculous meningoencephalitis alongside the discovery of an anterior mediastinal mass of tubercular origin, posed significant challenges to treatment. However, she ultimately experienced successful outcomes and recovered following surgery and medical treatment. Therefore, it is important to consider tuberculosis as a diagnosis when a patient presents with a mass in the anterior mediastinum, despite its rarity.
